# Alterations in brain function in patients with post-stroke cognitive impairment: a resting-state functional magnetic resonance imaging study

**DOI:** 10.3389/fnagi.2025.1501082

**Published:** 2025-02-19

**Authors:** Kaiyue Han, Linghui Dong, Xingxing Liao, Junzi Long, Jiarou Chen, Haitao Lu, Hao Zhang

**Affiliations:** ^1^School of Rehabilitation, Capital Medical University, Beijing, China; ^2^Beijing Bo’ai Hospital, China Rehabilitation Research Center, Beijing, China; ^3^Cheeloo College of Medicine, Shandong University, Jinan, China; ^4^Changping Laboratory, Beijing, China; ^5^The Second School of Medicine, Wenzhou Medical University, Wenzhou, China; ^6^University of Health and Rehabilitation Sciences, Qingdao, China

**Keywords:** stroke, post-stroke cognitive impairment, resting-state functional magnetic resonance imaging, fractional amplitude of low-frequency fluctuations, regional homogeneity, functional connectivity

## Abstract

**Background:**

Cognitive impairment is a common dysfunction following stroke, significantly affecting patients’ quality of life. Studies suggest that post-stroke cognitive impairment (PSCI) may be related to neural activity in specific brain regions. However, the neural mechanisms remain to be further explored. This study aimed to investigate the alterations in brain function in patients with PSCI.

**Methods:**

This was a case–control study. Thirty patients with PSCI, thirty with non-PSCI (NPSCI), and thirty age- and gender-matched healthy controls (HCs) were selected in a 1:1:1 ratio. Resting-state functional magnetic resonance imaging (rs-fMRI) were acquired from all participants to study the potential neural mechanisms of PSCI patients by comparing the differences in fractional amplitude of low-frequency fluctuation (fALFF), Kendall’s coefficient of concordance-regional homogeneity (KCC-ReHo), and seed-based functional connectivity (FC). Additionally, the Montreal Cognitive Assessment (MoCA) scores of PSCI patients were collected, and Pearson correlation was used to analyze the correlation between functional indicators and cognitive performance in PSCI patients.

**Results:**

fALFF analysis revealed that the PSCI group had decreased zfALFF values in the left caudate, right inferior temporal gyrus (ITG), anterior cingulate cortex (ACC), left putamen, and left superior temporal gyrus. In contrast, increased zfALFF values were observed in the right Cerebellum_6. KCC-ReHo analysis indicated that the PSCI group had decreased SzKCC-ReHo values in the right middle frontal gyrus (MFG) and left postcentral lobe, while increased SzKCC-ReHo values in the left cerebellum_ crus 1, and left cerebellum_4–5. Furthermore, seed-based FC analysis revealed decreased zFC values between brain regions in the PSCI group, especially between the angular gyrus and precuneus. Additionally, correlation analysis showed that the zfALFF value of ACC was positively correlated with MoCA scores in the PSCI group.

**Conclusion:**

This study demonstrated significant changes in the spontaneous neural activity intensity, regional homogeneity, and FC of multiple cognition-related brain regions in PSCI patients, shedding light on the underlying neural mechanisms of brain function in PSCI.

## 1 Introduction

Stroke is one of the leading causes of long-term disability and death worldwide. Consequently, the structural damage it induces can have widespread effects on brain function beyond the focal lesion site (Griffis et al., 2020). Post-stroke cognitive impairment (PSCI) is one of the common sequelae among stroke survivors and can increase the risk of stroke recurrence (Sun et al., 2014). Notably, PSCI manifests as impairments in multiple cognitive domains, including memory, attention, language, and executive function (Lo et al., 2019). However, the neural mechanisms underlying PSCI remain unclear. Importantly, previous studies have demonstrated that abnormal neural activity and functional connectivity (FC) in brain regions related to stroke may be one of the primary mechanisms (Lim and Kang, 2015).

The neurological symptoms of PSCI have provided an important basis for understanding the brain map related to cognition. Previous studies have also enhanced our knowledge of various cognitive domain functions and the associated brain region localization (Broca, 1863; Ladino et al., 2018). Although stroke location is one of the most critical determinants of cognitive outcomes (Munsch et al., 2016), the development of brain network research suggests that cognitive dysfunctions cannot be entirely explained by it alone. Some higher cognitive functions, such as memory and execution, appear to depend on communication among multiple brain regions rather than one specific region (Wernicke, 1969). Functional magnetic resonance imaging (fMRI) is a widely used technique for studying brain functional in neurological and psychiatric diseases (Sun et al., 2011). One specific type, resting-state fMRI (rs-fMRI) technique, measures temporal correlation of variations in blood oxygen level-dependent (BOLD) signal across brain regions in a quiet state. Notably, it measures temporal coherence between brain regions (Azeez and Biswal, 2017).

Stroke leads to changes in the intensity of spontaneous neural activity in local brain regions. It has been shown that neural activity of certain brain regions in PSCI patients in the resting state exhibits significant differences compared to healthy individuals (Lim et al., 2021). In particular, regions associated with higher cognitive functions, such as the prefrontal cortex, hippocampus, and cingulate gyrus, often show abnormal activity in PSCI patients (Price et al., 2017). Functional alterations in these brain regions may reflect brain tissue damage, neuronal loss, and compensatory neural activity after stroke (Dubovik et al., 2012). To better understand changes in brain activity, Zang et al. (2007) proposed an amplitude of low-frequency fluctuation (ALFF) calculation method to reflect the intensity of spontaneous neural activity in local brain regions, referring to the sum of signal spectral amplitudes of all voxels in the low-frequency range. However, ALFF is easily affected by general physiological noise, so Zou et al. (2008) proposed another fractional amplitude of low-frequency fluctuation (fALFF) calculation method, which is a ratio of the ALFF to the sum of amplitudes in the full frequency range. From previous studies, the presence of stroke sites leads to changes in fALFF in local brain regions of PSCI patients (Wang S. et al., 2021). Still, the specific mechanisms of changes remain unclear, particularly regarding the influence of stroke lesion variability, neuroplasticity, and other potential confounding factors.

Damage to specific brain regions resulting from stroke can harm local and global function (Dijkhuizen et al., 2014). KCC-ReHo (Kendall’s coefficient of concordance-regional homogeneity) and FC analysis are commonly used methods for evaluating brain function (Zhang et al., 2022), especially with FC playing an essential role in understanding the neural mechanisms of PSCI. The human brain is a complex dynamic system. Notably, FC can quantify the temporal correlation of neurophysiological events in spatially different brain regions, revealing functional interactions between damaged and other brain areas (Auer, 2008). Studies have also found significant FC changes among PSCI patients’ brain regions (Carter et al., 2010). For example, PSCI patients with right hemisphere damage exhibit hemineglect and decreased interhemispheric FC in the parietal cortex. These changes may be due to stroke-induced neural pathway damage and reconnection, affecting the coordination and information processing abilities among different brain regions (Grefkes and Fink, 2014).

Stroke leads to focal, location-dependent neurological symptoms and affects different regions of affected and unaffected hemispheres through FC. Despite growing evidence highlighting the role of various brain regions in cognitive behavioral control, the neural mechanisms underlying brain function in PSCI patients remain unclear. Our study utilized rs-fMRI to provide neuroimaging evidence for the clinical diagnosis and effective treatment of PSCI. It aimed to further elucidate the neural mechanisms underlying brain function in PSCI by comparing alterations in zfALFF, SzKCC-ReHo, and seed-based zFC among PSCI, Non-PSCI (NPSCI), and healthy control (HC) groups.

## 2 Materials and methods

### 2.1 Participants

Participants were selected at the Neurorehabilitation Center of China Rehabilitation Research Center (CRRC) from March 2021 to August 2024. Strictly adhering to the following criteria, the study included thirty patients with PSCI as the experimental group, thirty patients with NPSCI and thirty age- and gender-matched healthy controls (HCs) as the control groups. This study was approved by the Medical Ethics Committee of the CRRC (No. 2024-086-02) and registered in the Chinese Clinical Trial Registry (ChiCTR2400089609).

For participants in the PSCI and NPSCI groups, who were all stroke patients, the shared inclusion criteria were as follows: (1) aged 35–75 years; (2) met diagnostic criteria of ischemic or hemorrhagic stroke (Warner et al., 2019; Greenberg et al., 2022), and was confirmed by computerized tomography (CT) or magnetic resonance imaging (MRI); (3) first-ever stroke, or stroke without significant sequelae; (4) stroke duration from 3 to 12 months; (5) stroke location in the unilateral supratentorial region; and (6) signed informed consent. Additionally, inclusion criteria for PSCI patients were also (1) met the PSCI diagnostic criteria (Wang K. et al., 2021); and (2) had mild to moderate cognitive impairment: the Mini-Mental State Examination (MMSE) < 27 or the Montreal Cognitive Assessment (MoCA) < 26, and MMSE ≥10. In contrast, the inclusion criteria for NPSCI patients were the MMSE ≥27 or the MoCA ≥26.

The shared exclusion criteria for stroke patients were as follows: (1) history of drug or alcohol abuse or substance misuse; (2) presence of severe depression, anxiety, or other psychiatric disorders; (3) presence of severe primary diseases in the circulatory, respiratory, digestive, urinary, endocrine, or hematopoietic systems that conventional medications cannot control; (4) presence of malignant hypertension or malignant tumors; (5) presence of severe infections, water and electrolyte imbalances, or acid–base disturbances; (6) presence of contraindications to MRI scans, such as any implanted metallic foreign bodies, electronic devices or claustrophobia; (7) pregnancy, breastfeeding, or planning to become pregnant; (8) concurrent participating in other clinical trials; and (9) presence of other conditions, as determined by the investigator, renders participants ineligible for the trial. Additionally, exclusion criteria for PSCI were also cognitive impairment resulting from not stroke, such as Alzheimer’s disease, frontotemporal dementia, lewy body dementia, vascular dementia, Parkinson’s disease dementia, traumatic brain injury, or other conditions.

For the HCs, the inclusion criteria were as follows: (1) aged 35–75 years; (2) physically healthy, with no acute or chronic diseases; (3) no history of major surgery or severe trauma; and (4) signed informed consent. The exclusion criteria were: (1) any current or past history of acute or chronic illnesses; (2) history of drug or alcohol abuse or substance misuse; (3) presence of severe depression, anxiety, or other psychiatric disorders; (4) presence of contraindications to MRI scans; (5) pregnancy, breastfeeding, or planning to become pregnant; (6) concurrent participating in other clinical trials; and (7) presence of other conditions, as determined by the investigator, renders participants ineligible for the trial.

### 2.2 Neuropsychological test

In this study, MoCA scores were collected from PSCI patients within 3 days after the rs-fMRI scan. MoCA is a rapid and validated tool for assessing cognitive function, including visuospatial and execution, naming, memory, attention, language, abstraction, and delayed recall (Shi et al., 2018). MoCA score ranges from 0 to 30, with higher scores indicating better cognitive function.

### 2.3 MRI data acquisition

Participants underwent MRI scanning using the same Philips Ingenia 3T MRI. To improve resolution, the scanner was equipped with a 32-channel head coil. Participants were instructed to close their eyes and stay awake during the scan. A rescan would be performed if significant head movement was detected during the scan, affecting data quality. Rs-fMRI images were collected with a gradient echo planar imaging (EPI) sequence: TR = 2000 ms, TE = 30 ms, field of view (FOV) = 224 × 224 mm^2^, flip angle (FA) = 90°, matrix = 64 × 64, number of slices = 32, and voxel size = 3.5 × 3.5 × 4.35 mm^3^. High-resolution T1-weighted images were acquired using a three-dimensional magnetization-prepared rapid gradient echo (3D-MPRAGE) sequence: TR = 7 ms, TE = 3.2 ms, FOV = 256 × 256 mm^2^, FA = 7°, matrix = 256 × 256 mm^2^, number of slices = 192, and voxel size = 1 × 1 × 1 mm^3^.

### 2.4 MRI data pre-processing

In the MATLAB environment (MathWorks, Natick, MA, USA), REST software (version 1.8)^1^ based on SPM 12^2^ was used to preprocess the rs-fMRI data and calculate brain functional indicators. Preprocessing procedures were as follows: (1) converting image files from EPI DICOM to NIFTI format; (2) removing the first 10 time points; (3) slice timing correction; (4) realigning correction for head motion, with the maximum head motion in x, y, and z directions being less than 2 mm and maximum angular rotation being less than 2°for all participants; (5) coregistering functional images with the corresponding 3D T1-weighted structural images, and then spatial normalized into the standard Montreal Neurological Institute (MNI) space with 3 × 3 × 3 mm^3^ resample; (6) spatial smoothing using a Gaussian kernel of a 6 mm full-width-at-half-maximum (FWHM); (7) removing linear trends; (8) performing band-pass filtering (0.01–0.08 Hz) to eliminate the effects of low-frequency drift and high-frequency noise (Biswal et al., 1995); and (9) regressing out nuisance covariates, including six head motion parameters, global mean, white matter, and cerebrospinal fluid signals (Friston et al., 1996).

The fALFF was a functional indicator reflecting the intensity of spontaneous neural activity in local brain regions. Unlike the methods above, the preprocessing of fALFF was not band-pass filtered. It transformed the time series of each voxel to the frequency domain for the power spectrum by a fast Fourier transform (FFT) and then calculated the square root of each frequency in the power spectrum, where the averaged square root across 0.01–0.08 Hz was the ALFF (Zang et al., 2007). Afterwards, the fALFF was calculated as the ratio of the ALFF to the sum of amplitudes in the full frequency range (Zou et al., 2008). Finally, to improve the normality and comparability of the data, a Fisher Z transformation was performed on the fALFF to obtain the zfALFF map.

KCC-ReHo evaluated this brain region’s functional homogeneity by measuring the time series’s coherence among neighboring voxels within that specific region (Zhang et al., 2022). The preprocessing of KCC-ReHo was not spatially smoothed. Instead, spatial smoothing was performed after obtaining KCC-ReHo values to derive SKCC-ReHo. Similarly, a Fisher Z transformation was performed on the SKCC-ReHo to obtain the SzKCC-ReHo map.

Seed-based correlation analysis was used to study the effect of local neural activity alterations on whole-brain FC. Clusters with significant differences among groups in fALFF were set as seeds. Each seed’s time series of each voxel was extracted in a spherical region (radius = 5 mm), and all voxels within the seed were averaged to obtain the mean time series of the seed region. Furthermore, Pearson correlation coefficients between the mean time series of each seed region and that of each voxel in the whole brain were then calculated, which were transformed into z-scores using Fisher r-to-z transformation to obtain the zFC maps.

### 2.5 Statistical analysis

Demographic and clinical variables of all participants were analyzed using SPSS statistics 26.0 (IBM Corp. Armonk, NY, USA). Shapiro–Wilk test was used to determine the normality of variable distribution. Normally distributed continuous variables were presented as means [standard deviation (SD)], and the comparisons between groups were conducted using one-way analysis of variance (ANOVA) or Student’s *t*-test. Categorical variables were presented as frequencies (percentages), and group comparisons were conducted using the chi-square or Fisher’s exact tests. The significance level was set at *p* < 0.05 (two-tailed).

One-way ANOVA was used to calculate fALFF, KCC-ReHo, and FC. Least Significant Difference (LSD) was used as *post hoc* analysis for the significant clusters of the inter-group differences [False discovery rate (FDR) correction, cluster-level *p* < 0.05, voxel-level *p* < 0.001, two-tailed]. Additionally, clusters with significant differences in fALFF values between the PSCI and NPSCI groups were selected, and Pearson correlation was used to analyze the correlation between the fALFF values of these clusters and MoCA scores in PSCI patients (*p* < 0.05, two-tailed).

## 3 Results

### 3.1 Demographic and clinical results

Table 1 showed no statistical differences in age, gender, and education level among the three groups (*p* > 0.05). Similarly, there were no statistical differences in stroke duration and type, lesion hemisphere, hypertension, hyperlipidemia, diabetes mellitus, smoking, alcoholism history, and National Institute of Health stroke scale (NHISS) scores (*p* > 0.05). On the contrary, there was a statistically significant difference in MMSE scores among the three groups (*p* < 0.001).

**Table 1:** The demographic and clinical characteristics.

Variables	PSCI (*n* = 30)	NPSCI (*n* = 30)	HC (*n* = 30)	*p-*value
Age, y, mean (SD)	51.97 (11.39)	53.07 (10.25)	47.57 (8.93)	0.095
Sex, male, n (%)	24 (80.0)	22 (73.3)	23 (76.7)	0.830
Education, y, mean (SD)	18.97 (4.22)	18.17 (3.46)	18.67 (4.36)	0.741
Stroke duration, d, mean (SD)	125.03 (38.65)	124.73 (44.99)	—	0.978
Stroke type, ischaemia, n (%)	11 (36.7)	16 (53.3)	—	0.194
Lesion hemisphere, left, n (%)	13 (43.3)	10 (33.3)	—	0.426
Hypertension, n (%)	26 (86.7)	21 (70.0)	18 (60.0)	0.066
Hyperlipidemia, n (%)	18 (60.0)	13 (43.3)	11 (36.7)	0.175
Diabetes mellitus, n (%)	13 (43.3)	11 (36.7)	9 (30.0)	0.563
Smoking history, n (%)	15 (50.0)	22 (73.3)	14 (46.7)	0.076
Alcoholism history, n (%)	15 (50.0)	17 (56.7)	23 (76.7)	0.088
NHISS, mean (SD)	6.03 (2.87)	5.07 (2.36)	—	0.160
MMSE, mean (SD)	21.57 (4.52)	28.87 (1.28)	29.77 (0.68)	<0.001

PSCI, post-stroke cognitive impairment; NPSCI, post-stroke without cognitive impairment; HC, healthy controls; MMSE, Mini-Mental State Examination; NHISS, National Institute of Health stroke scale; *p* < 0.05, statically different.

### 3.2 fALFF analysis

**Figure 1:**
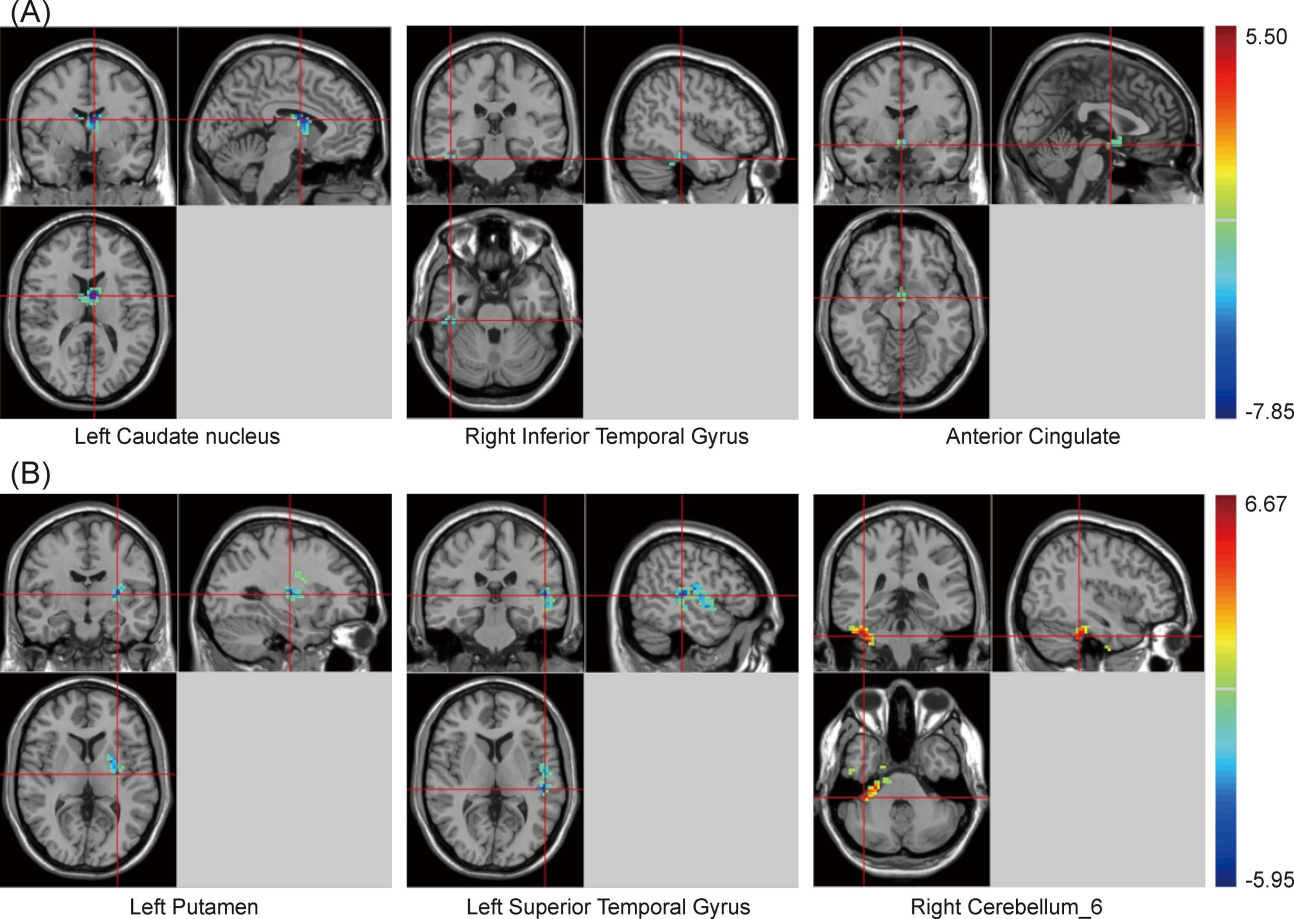
Brain maps of zfALFF differences. **(A)** PSCI vs. NPSCI. **(B)** PSCI vs. HC. zfALFF, z-score fractional amplitude of low-frequency fluctuations; PSCI, post-stroke cognitive impairment; NPSCI, non-PSCI; HC, healthy controls; Gaussian random field correction, cluster-level *p* < 0.05, voxel-level *p* < 0.001. The color bar represents *T* statistics. The red areas represent the regions which have increased zfALFF, while the blue ones represent the regions which have decreased zfALFF.

One-way ANOVA indicated significant differences in zfALFF values among the PSCI, NPSCI, and HC groups in the cingulate gyrus, putamen, inferior parietal lobe (IPL), left caudate, bilateral angular gyrus, and precuneus (Supplementary Table S1 and Figure 1). Further *post hoc* analysis was conducted with the PSCI group as the focus. Compared with the NPSCI group, the PSCI group exhibited decreased zfALFF values in the left caudate, right inferior temporal gyrus (ITG), and anterior cingulate cortex (ACC) (Table 2 and Figure 1A). Compared with the HC group, the PSCI group showed significantly decreased zfALFF values in the left putamen and left superior temporal gyrus (STG), while increased zfALFF values were observed in the right cerebellum_6 (Table 2 and Figure 1B).

**Table 2:** Statistical differences of zfALFF values between PSCI and NPSCI or HC groups.

Groups	Brain regions	Cluster	Peak MNI coordinates			Peak *t*-value
			X	Y	Z	
PSCI vs. NPSCI	Left Caudate Nucleus	29	−6	0	15	−7.85
	Right Inferior Temporal Gyrus	15	45	−27	−27	−5.81
	Anterior Cingulate	5	0	−3	−12	−5.43
PSCI vs. HC	Left Putamen	19	−30	−12	6	−5.31
	Left Superior Temporal Gyrus	16	−51	−27	6	−5.40
	Right Cerebellum_6	25	39	−36	−36	6.15

zfALFF, z-score frequency amplitude of low-frequency fluctuations; PSCI, post-stroke cognitive impairment; NPSCI, non-PSCI; HC, healthy controls; MNI, Montreal Neurological Institute; Gaussian random field correction, cluster-level *p* < 0.05, voxel-level *p* < 0.001.

### 3.3 KCC-ReHo analysis

**Figure 2:**
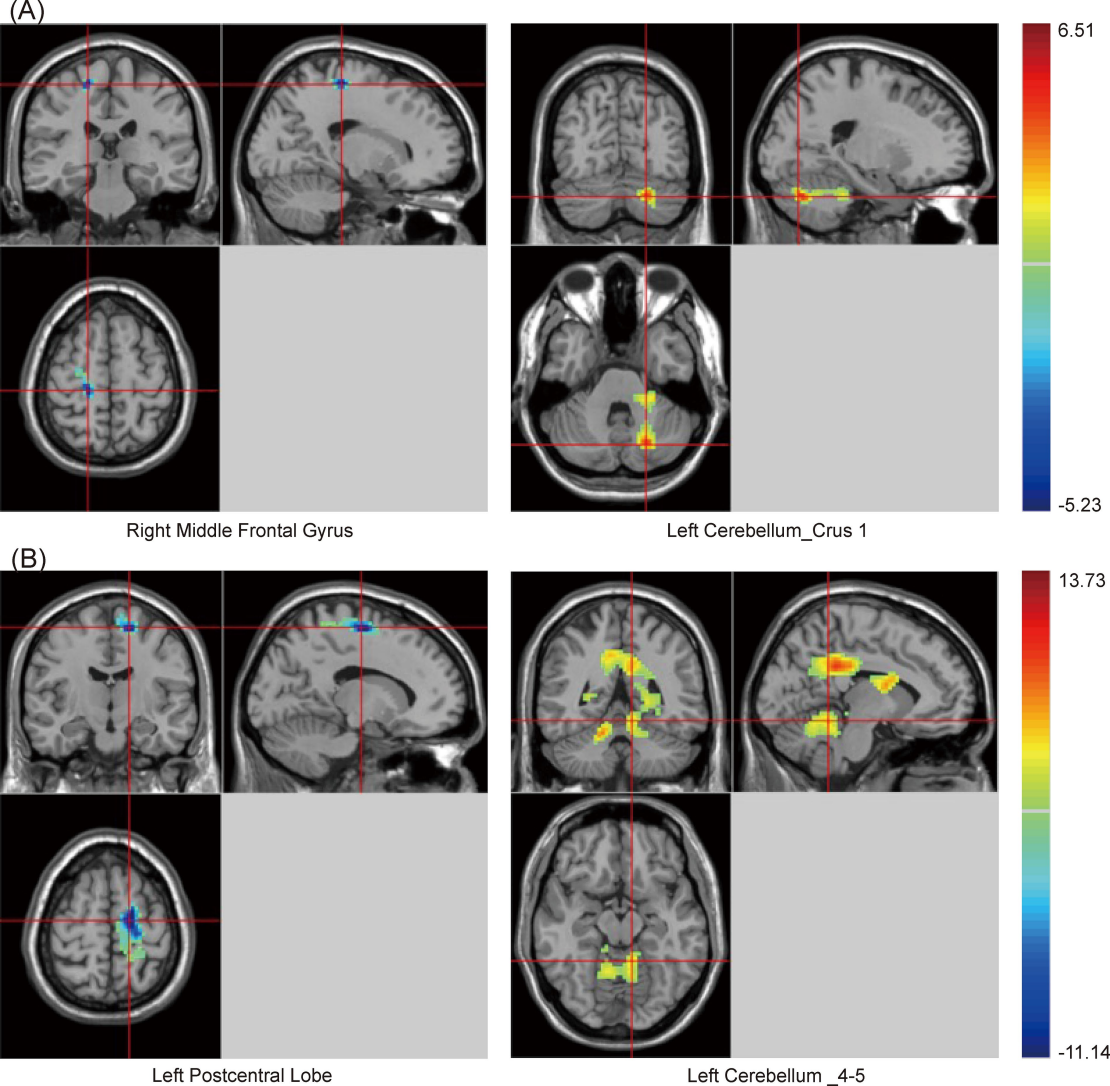
Brain maps of SzKCC-ReHo differences. **(A)** PSCI vs. NPSCI. **(B)** PSCI vs. HC. SzKCC-ReHo, Smoothed z-score Kendall’s Coefficient of Concordance based Regional Homogeneity; PSCI, post-stroke cognitive impairment; NPSCI, non-PSCI; HC, healthy controls; Gaussian random field correction, cluster-level *p* < 0.05, voxel-level *p* < 0.001. The color bar represents *T* statistics. The red areas represent the regions which have increased SzKCC-ReHo, while the blue ones represent the regions which have decreased SzKCC-ReHo.

Similarly, there were significant differences in SzKCC-ReHo values among the three groups (Supplementary Table S2 and Figure 2). *Post hoc* analysis with the PSCI group as the focus revealed that, compared with the NPSCI group, the PSCI group exhibited decreased SzKCC-ReHo values in the right middle frontal gyrus (MFG), while increased SzKCC-ReHo values in the left cerebellum_crus 1 (Table 3 and Figure 2A). Compared with the HC group, the PSCI group showed decreased SzKCC-ReHo values in the left postcentral lobe, while increased SzKCC-ReHo values were observed in the left cerebellum_4–5 (Table 3 and Figure 2B).

**Table 3:** Statistical differences of SzKCC-ReHo values between PSCI and NPSCI or HC groups.

Groups	Brain regions	Cluster	Peak MNI coordinates			Peak *t*-value
			X	Y	Z	
PSCI vs. NPSCI	Right Middle Frontal Gyrus	44	18	−27	60	−5.23
	Left Cerebellum_Crus 1	68	−21	−72	−33	5.64
PSCI vs. HC	Left Postcentral Lobe	89	−15	−12	63	−10.98
	Left Cerebellum _4–5	114	−9	−47	−14	11.69

SzKCC-ReHo, Smoothed z-score Kendall’s Coefficient of Concordance based Regional Homogeneity; PSCI, post-stroke cognitive impairment; NPSCI, non-PSCI; HC, healthy controls; MNI, Montreal Neurological Institute; Gaussian random field correction, cluster-level *p* < 0.05, voxel-level *p* < 0.001.

### 3.4 Seed-based FC analysis

**Figure 3:**
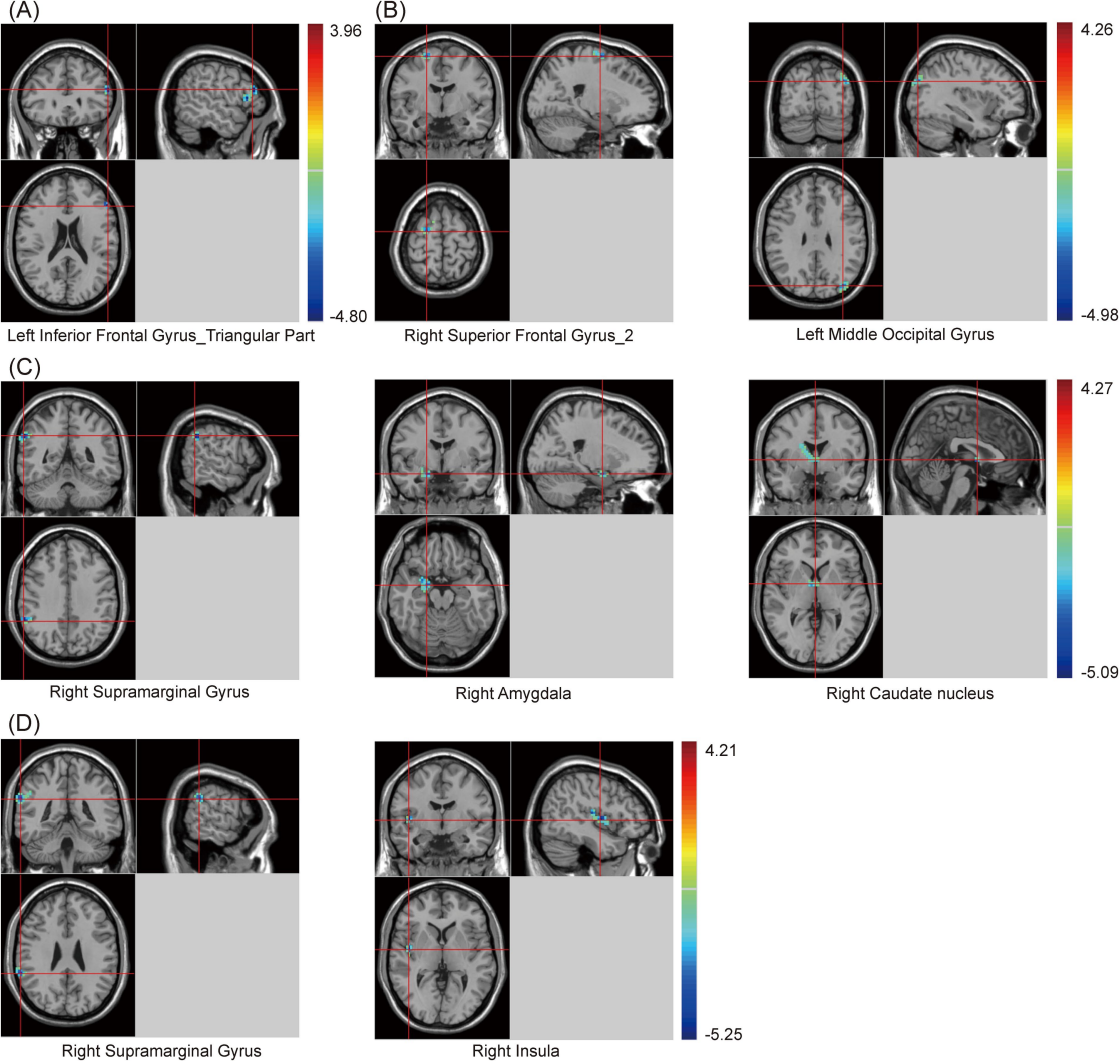
Brain maps of seed-based zFC differences between PSCI and NPSCI. **(A)** The seed is cingulate gyrus. **(B)** The seed is right angular gyrus. **(C)** The seed is left angular gyrus. **(D)** The seed is precuneus. zFC, z-score functional connectivity; PSCI, post-stroke cognitive impairment; NPSCI, non-PSCI; Gaussian random field correction, cluster-level *p* < 0.05, voxel-level *p* < 0.001. The color bar represents *T* statistics. The red areas represent the regions which have increased zFC, while the blue ones represent the regions which have decreased zFC.

In this study, clusters with significant differences in zfALFF values among the three groups were set as seeds, including the cingulate gyrus, right angular gyrus (RAG), left angular gyrus (LAG), and precuneus. The results showed that, regardless of which seed was used for the zFC analysis, there were significant differences among the three groups (Supplementary Table S3 and Figure 3). Similarly, *post hoc* analysis with the PSCI group as the focus was conducted. Compared with the NPSCI group, when using the cingulate gyrus as the seed, the zFC value between the cingulate gyrus and the left inferior frontal gyrus (IFG)_triangular part decreased in the PSCI group. When using RAG as the seed, the zFC values between the RAG and two clusters, including the right superior frontal gyrus (SFG)_2 and left middle occipital gyrus (MOG), decreased in the PSCI group. When using LAG as the seed, the zFC values between the LAG and three clusters, including the right supramarginal gyrus (SMG), right amygdala, and right caudate, decreased in the PSCI group. When using the precuneus as the seed, the zFC values between the precuneus and two clusters, including the right SMG and right insula, decreased in the PSCI group (Table 4 and Figure 3).

**Table 4:** Statistical differences of seed-based zFC values between PSCI and NPSCI groups.

Seeds	Brain regions	Cluster	Peak MNI coordinates			Peak *t*-value
			X	Y	Z	
Cingulate Gyrus	Left Inferior Frontal Gyrus_Triangular Part	42	−54	30	21	−4.80
Right Angular Gyrus	Right Superior Frontal Gyrus_2	36	21	−6	66	−4.36
	Left Middle Occipital Gyrus	35	−36	−81	30	−4.18
Left Angular Gyrus	Right Supramarginal Gyrus	44	60	−48	36	−5.07
	Right Amygdala	22	21	−3	−18	−4.22
	Right Caudate nucleus	18	1	0	2	−4.24
Precuneus	Right Supramarginal Gyrus	39	63	−42	27	−4.68
	Right Insula	25	45	−6	3	−5.25

zFC, z-score functional connectivity; PSCI, post-stroke cognitive impairment; NPSCI, non-PSCI; HC, healthy controls; MNI, Montreal Neurological Institute; Gaussian random field correction, cluster-level *p* < 0.05, voxel-level *p* < 0.001.

**Figure 4:**
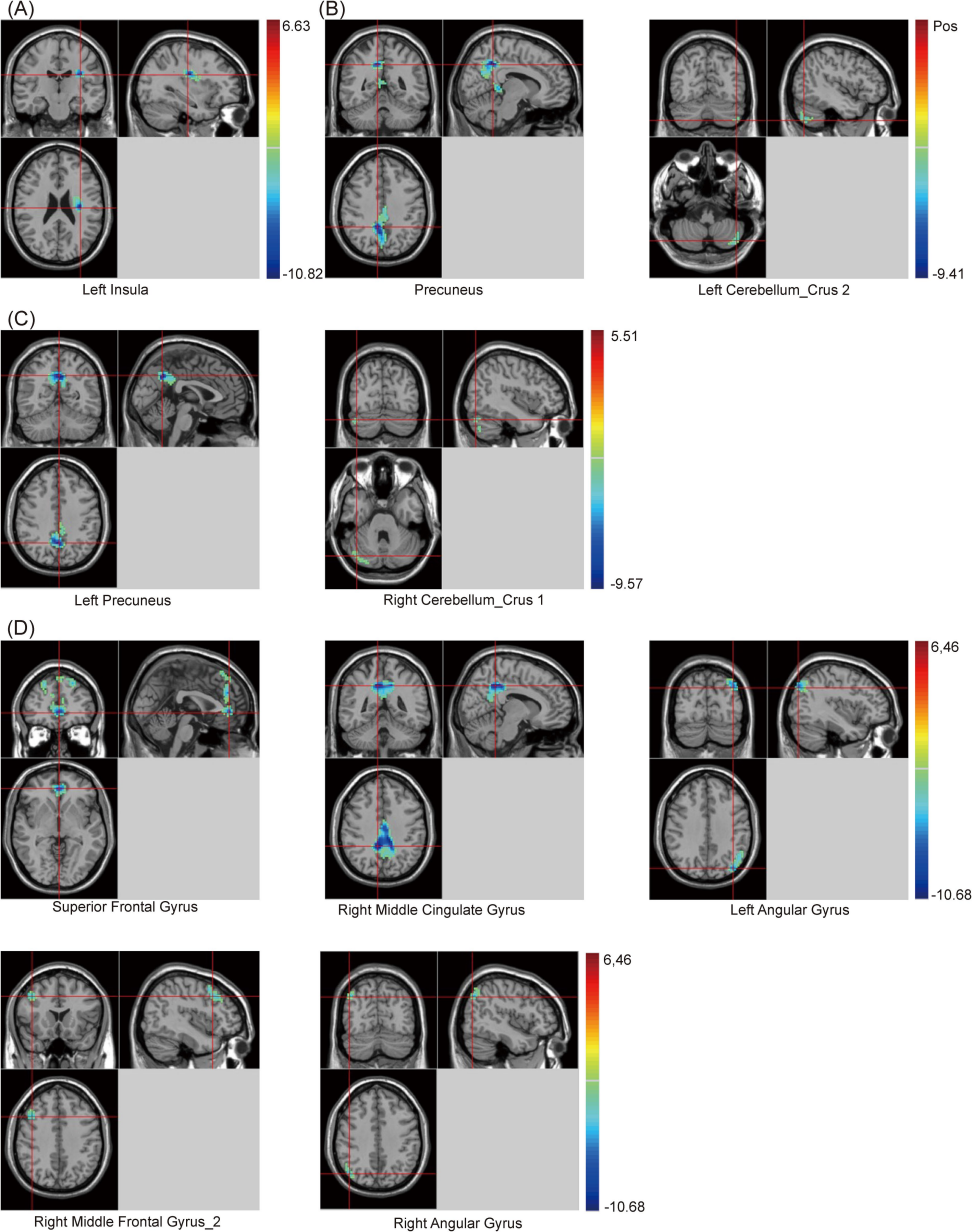
Brain maps of seed-based zFC differences between PSCI and HC. **(A)** The seed is cingulate gyrus. **(B)** The seed is right angular gyrus. **(C)** The seed is left angular gyrus. **(D)** The seed is precuneus. FC, z-score functional connectivity; PSCI, post-stroke cognitive impairment; HC, healthy controls; Gaussian random field correction, cluster-level *p* < 0.05, voxel-level *p* < 0.001. The color bar represents *T* statistics. The red areas represent the regions which have increased zFC, while the blue ones represent the regions which have decreased zFC.

Additionally, compared with the HC group, when using the cingulate gyrus as the seed, the zFC value between the cingulate gyrus and left Insula decreased in the PSCI group. When using RAG as the seed, the zFC values between the RAG and two clusters, including the precuneus and left cerebellum_crus 2, decreased in the PSCI group. When using LAG as the seed, the zFC values between the LAG and two clusters, including the left precuneus and right cerebellum_crus 1, decreased in the PSCI group. Furthermore, when using the precuneus as the seed, the zFC values between the precuneus and five clusters, including the SFG, right middle cingulate gyrus (MCG), bilateral angular gyrus, and right MFG_2, decreased in the PSCI group (Table 5 and Figure 4).

**Table 5:** Statistical differences of seed-based zFC values between PSCI and HC groups.

Seeds	Brain regions	Cluster	Peak MNI coordinates			Peak *t*-value
			X	Y	Z	
Cingulate Gyrus	Left Insula	13	−33	−18	24	−10.82
Right Angular Gyrus	Precuneus	306	9	−48	39	−9.41
	Left Cerebellum_Crus 2	45	−45	−69	−48	−6.45
Left Angular Gyrus	Left Precuneus	102	−1	−57	39	−9.57
	Right Cerebellum_Crus 1	34	42	−75	−30	−6.30
Precuneus	Superior Frontal Gyrus	304	0	45	−3	−9.08
	Right Middle Cingulate Gyrus	205	9	−45	39	−10.68
	Left Angular Gyrus	111	−39	−78	36	−7.96
	Right Middle Frontal Gyrus_2	82	42	18	39	−7.00
	Right Angular Gyrus	73	45	−72	39	−7.37

zFC, z-score functional connectivity; PSCI, post-stroke cognitive impairment; NPSCI, non-PSCI; HC, healthy controls; MNI, Montreal Neurological Institute; Gaussian random field correction, cluster-level *p* < 0.05, voxel-level *p* < 0.001.

### 3.5 Correlation analysis

**Figure 5:**
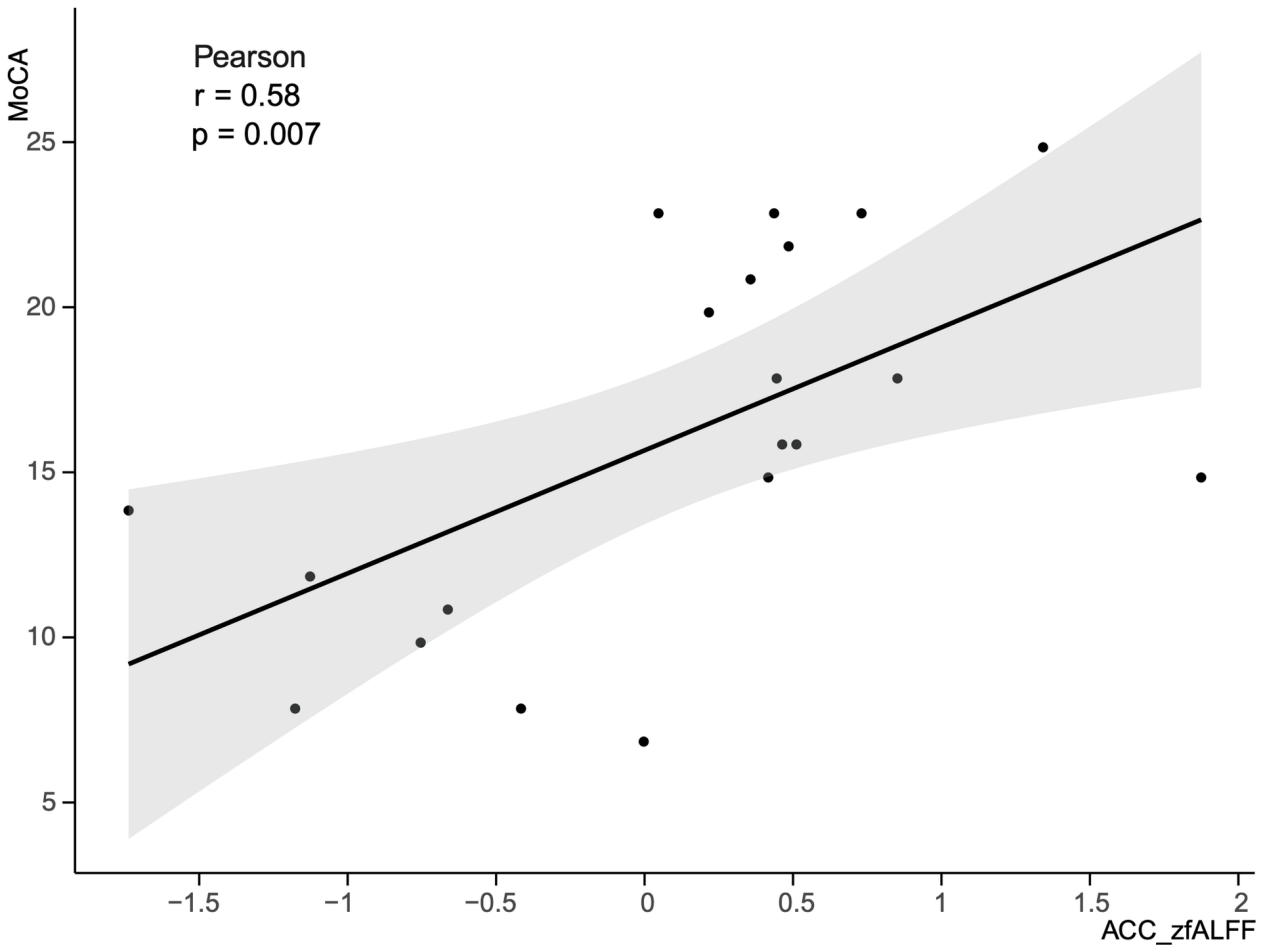
Correlation between the zfALFF value of the ACC and MoCA scores in PSCI group. zfALFF, z-score fractional amplitude of low-frequency fluctuations; ACC, Anterior Cingulate cortex; MoCA, Montreal Cognitive Assessment Scale; PSCI, post-stroke cognitive impairment.

This study selected clusters with significant differences in zfALFF values between the PSCI and NPSCI groups, including the left caudate, left precentral gyrus, right ITG, and ACC, to explore the relationship between fMRI and cognition. We calculated the correlation between the zfALFF values of four clusters and the MoCA scores of PSCI patients separately. The results showed that only the zfALFF value of the ACC was positively correlated with the MoCA scores (Figure 5).

## 4 Discussion

PSCI may be related to factors such as the intensity of spontaneous neural activity at the stroke site, the neural activity synchrony with nearby brain regions, and alterations in FC patterns between different brain regions. In this study, our results showed significant differences in zfALFF, SzKCC-ReHo, and seed-based zFC between PSCI and NPSCI or HC groups.

### 4.1 fALFF analysis

In the resting state, the PSCI group exhibited decreased zfALFF values in the left caudate, right ITG, and ACC compared with the NPSCI group, which is likely related to cognitive control and emotional regulation roles of these regions. Recent studies have also shown that the caudate is critical for working memory and attention control processes (Chiu et al., 2017; Samrani and Persson, 2024), while the ITG is important for advanced visual processing and semantic processing (Nobre et al., 1994; Schweinberger et al., 2002). Moreover, Bush et al. (2000) indicated significant activity in the ACC during cognitive control and emotional conflict, and Fleck et al. (2006) highlighted ACC plays a vital role in decision-making processes. Our study also revealed a positive correlation between MoCA scores and zfALFF values of the ACC, suggesting that the spontaneous neural activity intensity in the ACC is associated with cognitive dysfunction. When compared with the HC group, the PSCI group demonstrated decreased zfALFF values in the left putamen and left STG, which are involved in motor learning (Lee and Seo, 2016; Haber and Knutson, 2010) and sensory processing (Alloway et al., 2017).

### 4.2 KCC-ReHo analysis

Alterations in the coherence of specific brain regions have been observed in patients with PSCI (Liu et al., 2014). Yuan and Raz (2014) found that the MFG exhibited decreased neural synchrony after impairments in executive function. Consistent with these studies, our findings showed that the PSCI group exhibited decreased KCC-ReHo values in the right MFG. Additionally, the PSCI group also showed increased KCC-ReHo values in certain cerebellar regions, including the left cerebellum_crus 1 and left cerebellum_4–5. The cerebellum is part of cognitive and emotional circuits (Stoodley et al., 2016), and its increased synchrony may reflect compensatory mechanisms in response to cognitive decline. Wu et al. (2022) also demonstrated increased ReHo values in the cerebellum_4–5 in patients with mild cognitive impairment, further highlighting the cerebellum’s role in cognitive function.

### 4.3 Seed-based FC analysis

Previous studies found that PSCI patients diminished FC in the default mode network (DMN), with significant changes also observed in the ACC and precuneus (Yue et al., 2023). In our study, when the cingulate gyrus was used as the seed, the PSCI group showed significantly decreased zFC values between the cingulate gyrus and the left IFG_triangular part, as well as the left insula. These findings suggested altered interactions between these regions, which are involved in cognitive control and emotional regulation (Seamans and Floresco, 2022). Notably, the weakened FC could contribute to cognitive deficits in PSCI patients, particularly in executive function (Dosenbach et al., 2006).

The PSCI group showed significant alterations in zFC values among multiple brain regions when using the angular gyrus as the seed. Among these, the zFC values were decreased between the RAG and several regions, including the right SFG_2, left MOG, precuneus, and left cerebellum_crus 2. These regions are crucial for cognitive functions, and decreased FC in these areas may reflect the cognitive decline observed in PSCI (Buckner et al., 2008). Furthermore, when the LAG was used as the seed, reduced zFC values in the PSCI group were also found between the LAG and several regions, such as the right SMG, right amygdala, right caudate, left precuneus, and right cerebellum_crus 1. These areas may be related to language processing, emotional regulation, and executive functions (Baghernezhad and Daliri, 2024). It is well known that the caudate and amygdala are part of the striatum, which belongs to the SN and is involved in memory and learning functions (Milardi et al., 2019). Additionally, Stoodley and Schmahmann (2009) indicated that the cerebellum plays a significant role in cognitive processing and emotional regulation, and weakened connectivity may be associated with the extensive cognitive deficits observed in PSCI. Furthermore, our study suggested that alterations in FC may be a crucial basis for cognitive dysfunction in PSCI patients.

Finally, when using the precuneus as the seed, the PSCI group also showed decreased zFC between the precuneus and several regions, such as the right SMG, right insula, SFG, and right MCG. The precuneus is a key node in the DMN and is involved in the limbic system. Thus, weakened connectivity in this area may reflect impairments in memory and spontaneous thinking in PSCI patients (Greicius et al., 2003; Cavanna and Trimble, 2006).

### 4.4 Limitations

This study provides new insights into the neural mechanisms of brain function in PSCI patients, but it also has some limitations. First, there was some heterogeneity in the clinical characteristics of the participants. However, due to the small sample size in each group, we did not divide the subgroups by stroke location or severity to discuss their effects on brain function separately. Second, this study was a case–control study that only observed inter-group differences at a specific time and did not dynamically observe brain functional alterations in PSCI patients over time. In the future, a larger sample size and long-term follow-up should be conducted to further elucidate the neural mechanisms of PSCI and to provide evidence-based support for the cognitive function recovery of PSCI patients.

## 5 Conclusion

This study revealed significant changes in spontaneous neural activity intensity, regional homogeneity, and FC of multiple cognition-related brain regions in PSCI patients, providing new insights into the neural mechanisms in PSCI. Future research should further explore the specific mechanisms of functional alterations in key brain regions in PSCI, offering theoretical support for clinical treatment strategies.

## Data Availability

The raw data supporting the conclusions of this article will be made available by the authors, without undue reservation.

## Footnotes

^1^ http://www.restfmri.net/

^2^ http://www.fil.ion.ucl.ac.uk/spm
