# Supplementary material for: Alterations in brain function in patients with post-stroke cognitive impairment: a resting-state functional magnetic resonance imaging study
Source: Front Aging Neurosci. 2025 Feb 19;17:1501082. doi: 10.3389/fnagi.2025.1501082 (PMC11880027; doi:10.3389/fnagi.2025.1501082)
Supplement: Supplementary file 1 [file Data_Sheet_1.docx]

Supplementary Material

# 1 Supplementary Tables

**Table S1 Statistical differences of zfALFF values between PSCI, NPSCI and HC groups.**

| **Brain regions** | **Cluster** | **Peak MNI coordinates** | | | **Peak *F*-value** |
| --- | --- | --- | --- | --- | --- |
|  |  | **X** | **Y** | **Z** |  |
| Cingulate Gyrus | 200 | -30 | -6 | 24 | 52.73 |
| Putamen | 102 |  |  |  |  |
| Inferior Parietal Lobe | 41 |  |  |  |  |
| Left Caudate Nucleus | 20 |  |  |  |  |
| Right Angular Gyrus | 138 | 51 | -66 | 33 | 46.09 |
| Left Angular Gyrus | 121 | -33 | -72 | 42 | 33.05 |
| Precuneus | 41 | 0 | -60 | 39 | 25.61 |

zfALFF, z-score frequency amplitude of low-frequency fluctuations; PSCI, post-stroke cognitive impairment; NPSCI, non-PSCI; HC, healthy controls; MNI, Montreal Neurological Institute; Gaussian random field correction, cluster-level *p* < 0.05, voxel-level *p* < 0.001.

**Table S2 Statistical differences of SzKCC-ReHo values between PSCI, NPSCI and HC groups.**

| **Brain regions** | **Cluster** | **Peak MNI coordinates** | | | **Peak**  ***F*-value** |
| --- | --- | --- | --- | --- | --- |
|  |  | **X** | **Y** | **Z** |  |
| Cerebellum Anterior Lobe | 81 | 12 | -33 | -24 | 30.98 |
| Left Caudate | 69 | -18 | -30 | 15 | 60.30 |
| Left Cerebellum _4-5 | 59 | -15 | -42 | -24 | 33.76 |
| Left Superior Parietal Lobe | 31 | -18 | -54 | 63 | 25.62 |
| Right Supramarginal Gyrus | 30 | 57 | -24 | 21 | 30.73 |
| Left Postcentral Lobe | 21 | -54 | -18 | 24 | 28.51 |

SzKCC-ReHo, Smoothed z-score Kendall's Coefficient of Concordance based Regional Homogeneity; PSCI, post-stroke cognitive impairment; NPSCI, non-PSCI; HC, healthy controls; MNI, Montreal Neurological Institute; Gaussian random field correction, cluster-level *p* < 0.05, voxel-level *p* < 0.001.

**Table S3 Statistical differences of seed-based zFC values between PSCI, NPSCI and HC groups.**

| **Seeds** | **Brain regions** | **Cluster** | **Peak MNI coordinates** | | | **Peak**  ***F*-value** |
| --- | --- | --- | --- | --- | --- | --- |
|  |  |  | **X** | **Y** | **Z** |  |
| Cingulate Gyrus | Left Superior Medial Frontal Gyrus | 37 | -3 | 66 | 21 | 11.46 |
|  | Left Insula | 22 | -33 | -15 | 24 | 59.64 |
| Right Angular Gyrus | Precuneus | 266 | -12 | -66 | 30 | 19.06 |
|  | Right Middle Frontal Gyrus_2 | 144 | 60 | 18 | 27 | 23.95 |
|  | Left Cerebellum_Crus 2 | 98 | -48 | -75 | -39 | 20.27 |
|  | Supramarginal Gyrus | 80 | 48 | -69 | 39 | 39.83 |
|  | Right Inferior Parietal Lobe | 40 |  |  |  |  |
|  | Left Middle Occipital Gyrus | 38 | -51 | -69 | 42 | 18.61 |
|  | Left Inferior Parietal Lobe | 10 |  |  |  |  |
| Left Angular Gyrus | Left Precuneus | 213 | 0 | -57 | 36 | 49.02 |
|  | Right Precuneus | 177 |  |  |  |  |
|  | Left Middle Frontal Gyrus_2 | 84 | -42 | 27 | 36 | 19.50 |
|  | Right Cerebellum_Crus 1 | 80 | 33 | -84 | -30 | 22.42 |
|  | Left Inferior Temporal Gyrus | 37 | -63 | -30 | -18 | 22.12 |
| Precuneus | Superior Frontal Gyrus | 509 | 21 | 27 | 51 | 40.57 |
|  | Posterior Cingulate | 249 | -9 | -39 | 39 | 57.37 |
|  | Left Angular Gyrus | 129 | -36 | -78 | 42 | 39.12 |
|  | Right Middle Frontal Gyrus_2 | 112 | 42 | 21 | 36 | 30.69 |
|  | Left Middle Temporal Gyrus | 62 | -66 | -48 | 0 | 21.19 |
|  | Left Pre-Anterior Cingulate Cortex | 48 | 0 | 45 | -3 | 31.55 |
|  | Right Pre-Anterior Cingulate Cortex | 21 |  |  |  |  |

zFC, z-score functional connectivity; PSCI, post-stroke cognitive impairment; NPSCI, non-PSCI; HC, healthy controls; MNI, Montreal Neurological Institute; Gaussian random field correction, cluster-level *p* < 0.05, voxel-level *p* < 0.001.

# 2 Supplementary Figures


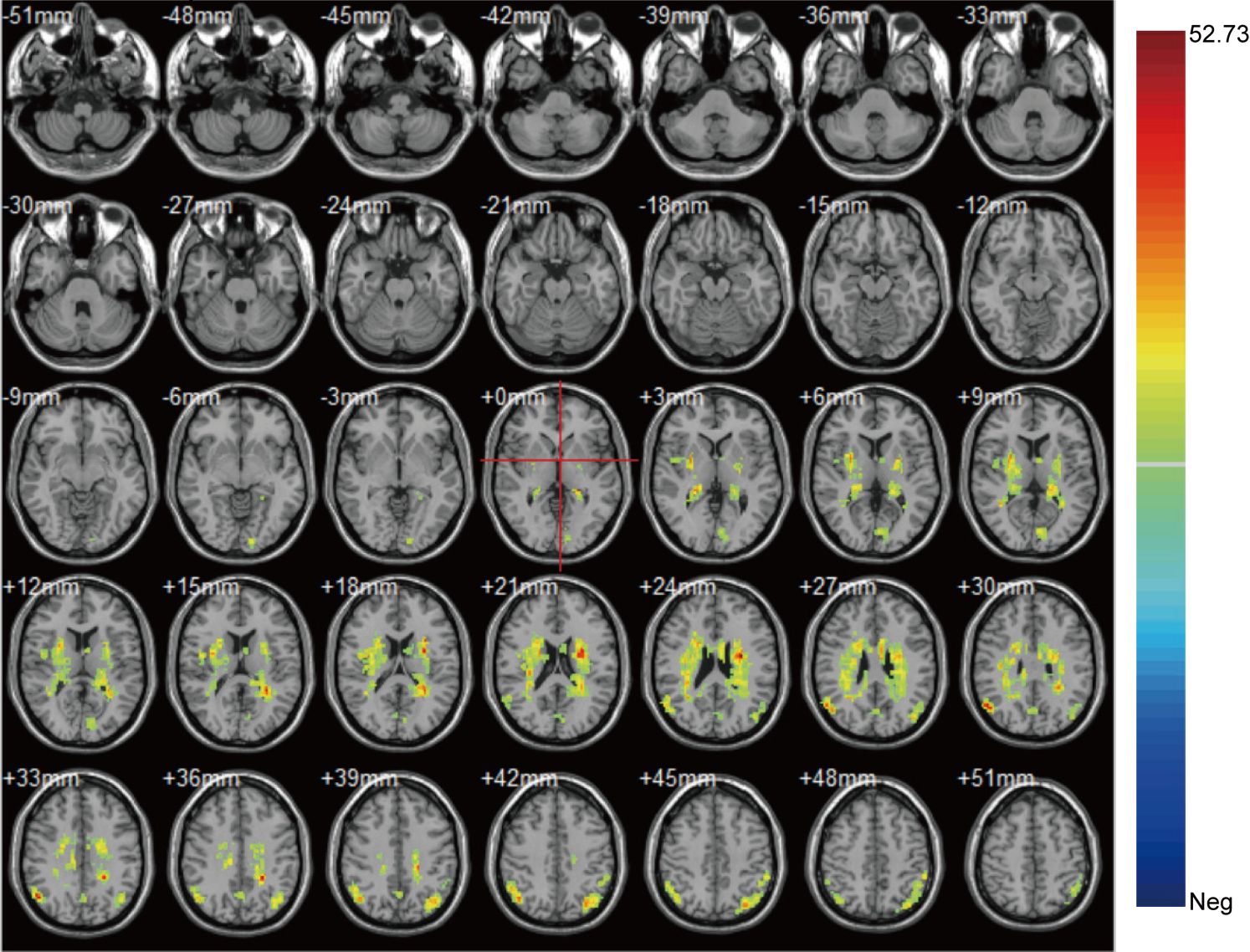


**Figure S1**. Brain maps of zfALFF differences between PSCI, NPSCI and HC group. zfALFF, z-score fractional amplitude of low-frequency fluctuations; PSCI, post-stroke cognitive impairment; NPSCI, non-PSCI; HC, healthy controls; Gaussian random field correction, cluster-level *p* < 0.05, voxel-level *p* < 0.001. The color bar represents *T* statistics. The red areas represent the regions which have increased zfALFF, while the blue ones represent the regions which have decreased zfALFF.


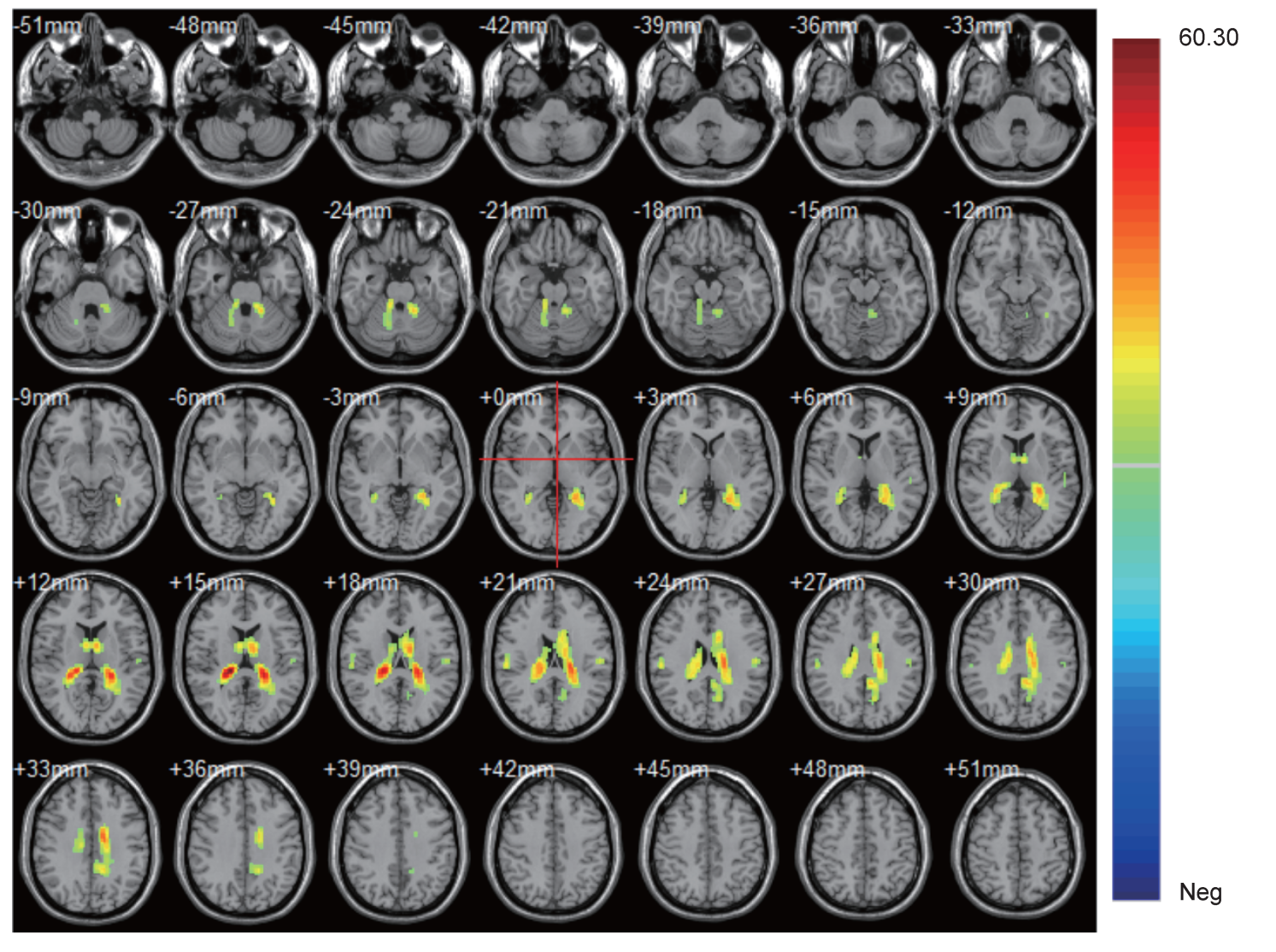


**Figure S2**. Brain maps of SzKCC-ReHo differences between PSCI, NPSCI and HC group. SzKCC-ReHo, Smoothed z-score Kendall's Coefficient of Concordance based Regional Homogeneity; PSCI, post-stroke cognitive impairment; NPSCI, non-PSCI; HC, healthy controls; Gaussian random field correction, cluster-level *p* < 0.05, voxel-level *p* < 0.001. The color bar represents *T* statistics. The red areas represent the regions which have increased SzKCC-ReHo, while the blue ones represent the regions which have decreased SzKCC-ReHo.

**
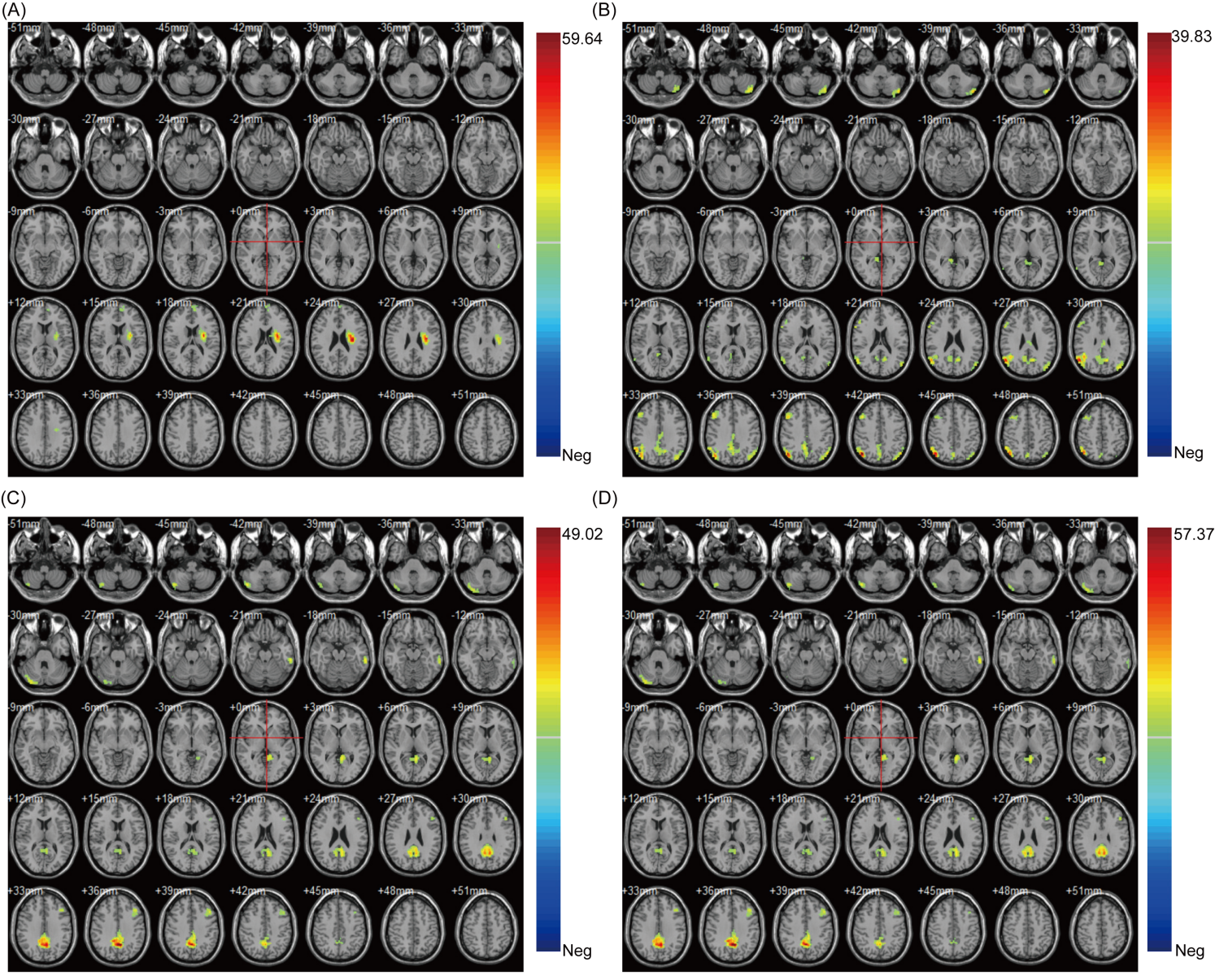
**

**Figure S3**. Brain maps of seed-based zFC differences between PSCI, NPSCI and HC group. **(A)** The seed is cingulate gyrus. **(B)** The seed is right angular gyrus. **(C)** The seed is left angular gyrus. **(D)** The seed is precuneus. zFC, z-score functional connectivity; PSCI, post-stroke cognitive impairment; NPSCI, non-PSCI; HC, healthy controls; Gaussian random field correction, cluster-level *p* < 0.05, voxel-level *p* < 0.001. The color bar represents *T* statistics. The red areas represent the regions which have increased zFC, while the blue ones represent the regions which have decreased zFC.
